# Involvement of P311 in the affective, but not in the sensory component of pain

**DOI:** 10.1186/1744-8069-4-23

**Published:** 2008-06-12

**Authors:** Yan-Gang Sun, Yong-Jing Gao, Zhong-Qiu Zhao, Bing Huang, Jun Yin, Gregory A Taylor, Zhou-Feng Chen

**Affiliations:** 1 Departments of Anesthesiology, Washington University School of Medicine Pain Center, St. Louis, Missouri 63110, USA; 2 Psychistry, Washington University School of Medicine, St. Louis, Missouri 63110, USA; 3 Developmental Biology, Washington University School of Medicine, St. Louis, Missouri 63110, USA; 4 Departments of Medicine, Molecular Genetics and Microbiology, and Immunology, Division of Geriatrics, and Center for the Study of Aging and Human Development, Duke University Medical Center, Durham, NC 27710, USA; 5 GRECC, VA Medical Center, Durham, NC 27705, USA; 6 Institute of Nautical Medicine, Nantong University, Nantong, 226001, PR China; 7 Department of Anesthesiology and Pain Management, Ministry of Health Beijing Hospital, Beijing, 100730, PR China

## Abstract

Pain is comprised of the sensory and affective components. Compared to the well-investigated mechanisms of the sensory pain, much less is known about the mechanisms underlying the affective pain. In recent years, accumulating evidence suggests that the anterior cingulate cortex (ACC) is a key structure for pain affection. To identify the molecules that may be involved in the affective component of pain, we have searched the Allen Brain Atlas expression database for genes whose expression is enriched in the ACC, and found that P311, an 8-kDa peptide, showed the strong expression in the ACC. P311 is also expressed in other areas associated with pain affection including the amygdala, insular cortex and thalamus. To understand the role of P311 in pain perception, we have examined the pain behaviors of the mice lacking P311. P311^-/- ^mice showed normal heat and mechanical sensitivity, as well as normal formalin-induced inflammatory pain. In contrast, the formalin-induced avoidance behavior, which reflects pain-related  negative emotion, was significantly attenuated in P311^-/- ^mice relative to the control mice. These results suggest that P311 is involved in the affective, but not in the sensory component of pain. Our study thus provides the first evidence suggesting that the affective and sensory pain may be regulated by distinct molecular mechanisms.

## Introduction

The pain experience includes a sensory-discriminative dimension, such as its location, intensity and quality, and an affective dimension, such as unpleasantness and emotions associated with future implications (secondary affect) [1-3]. Recent studies in humans and experimental animals have established that the sensory and affective dimensions of pain are processed by partially dissociable brain networks [2,3]. Human imaging studies showed that the activity of the somatosensory (SS) cortex is correlated with the intensity of the noxious heat, whereas that of the anterior cingulate cortex (ACC) with subjective unpleasantness [4,5], suggesting a differentially functional requirement for the SS cortex and ACC in pain perception [3]. Animal behavioral studies using the formalin-induced conditioned place avoidance (F-CPA) or place escape/avoidance paradigm also support the hypothesis that the ACC mediates the affective-like responses of tonic pain [6-9].

Electrophysiological studies on neuroplasticity in the ACC have also suggested an involvement of the ACC in pain-related information processing [10,11]. It has been shown that the ACC neurons respond to noxious stimuli [12]. Importantly, peripheral injury caused long-term potentiation (LTP) in the ACC [13]. LTP may be the mechanisms shared by both pain and memory [14]. LTP can be induced in the ACC, and numerous molecular pathways underlying the LTP in the ACC have been identified by genetic and pharmacological approaches [10,15,16]. In contrast to molecular mechanisms of the sensory component of pain which have been subjected to intensive studies [17], the molecular basis for pain affection remains elusive. To elucidate the molecular mechanisms underlying the affective component of pain, we identified several genes that are highly expressed in the ACC compared with the SS cortex, and examined one of the genes, P311, in pain affection.

P311 (also called PTZ17) was first identified because of its high expression in the embryonic brain and does not belong to any known family [18]. P311 encodes an 8-kDa polypeptide and contains three PEST-like domains subjected to rapid degradation by multiple proteolytic pathways [19]. As a non-secreted peptide, P311 is highly conserved across the species and is localized in both the cytoplasm and nucleus [19,20]. Previous studies have implicated P311 in the induction of ameboid-like migration [21], glioblastoma cell migration [22], and in the myogenesis of smooth muscle myogenesis [19]. In the brain, P311 has been associated with seizures because its expression altered when seizures was induced [23]. Moreover, P311 expression was found to be up-regulated in the axotomized motoneurons during axonal elongation, and its overexpression can further enhance axon elongation of neurons *in vivo *and *in vitro *[20]. In this report, we examined the role of P311 not only in the sensory but also in the affective component of pain processing in mice.

## Methods

### Identification of ACC higher genes

The ACC-enriched genes were identified by searching the Allen Brain Atlas (ABA) [24]. Total of 2265 genes expressed in the cortex, of which 763 have coronal section expression data. The expression images of each gene (0–1.0 mm anterior to Bregma) containing both the ACC and SS were retrieved from ABA. And the expression level of each gene in the ACC and the SS measured by the density (ACC: V_ACC_, SS: V_SS_.) was analyzed using a NIH ImageJ software by manually defining the ACC and SS region in every section (0 represents the lowest expression, 255 represents the highest expression). The difference (D) is calculated by D = V_ACC_-V_SS_, and the genes are sorted by descending of D value. The higher D value means the higher expression in the ACC. The top 10 genes with higher expression in the ACC were listed in Table 1.

**Table 1:** Top 10 ACC-enriched genes

**#**	**Symbol**	Name	**Access #**	Widely expressed?
1	Dkk3	dickkopf homolog 3 (Xenopus laevis)	NM 015814	Yes
2	Itpka	inositol 1,4,5-trisphosphate 3-kinase A	NM 146125	Yes
3	Marcksl1	MARCKS-like 1	NM 010807	No
4	P311	DNA segment, human D4S114	NM 053078	No
5	Etv1	ets variant gene 1	NM 007960	No
6	Adcyap1	adenylate cyclase activating polypeptide 1	NM 009625	No
7	Ctnnb1	catenin (cadherin associated protein), beta 1	NM 007614	Yes
8	Cacna1h	calcium channel, voltage-dependent, T type, alpha 1H subunit	NM 021415	Yes
9	Ubtf	upstream binding transcription factor, RNA polymerase I	NM 011551	Yes
10	Tspyl2	TSPY-like 2	NM 029836	Yes

### Animals

P311^-/- ^and wild-type littermate mice were used in behavior experiments (Taylor et al, manuscript submitted). Male mice aged between 8 and 12 weeks were acclimatized to the experimental room and used for behavioral tests by observers blinded to the genotype of the animals. All the experiments were performed in accordance with the guidelines of the National Institutes of Health and the International Association for the Study of Pain and were approved by the Animal Studies Committee at Washington University School of Medicine.

### Pain behavioral experiments

Pain behavior tests were performed as described [25,26]. Briefly, thermal sensitivity was determined using hot-plate (48, 52, 56°C), paw-flick (method of Hargreaves) or water immersion tail-flick methods (48, 50, 52°C). For the hot plate, the latency for the mouse to lick its hindpaw or jump was recorded. For the Hargreaves test, thermal sensitivity was measured using a Hargreaves-type apparatus (IITC Inc., Woodland Hills, CA), and the latency for the mouse to withdraw from the heat source was recorded. For water immersion tail-flick, tails dipped beneath the water in a temperature-controlled water bath (IITC Inc., Woodland Hills, CA), the latency to withdrawal was measured with a 10-s cutoff. Mechanical sensitivity: Mechanical sensitivity was assessed using a set of calibrated von Frey filaments (Touch-Test kit, Stoelting, Chicago, IL). Each filament was applied 5 consecutive times and the smallest filament that evoked reflexive flinches of the paw on 3 of the 5 trials was taken as paw withdrawal threshold. The formalin test was performed by intraplantar injection of formalin (Sigma, 15 μl of 5% formalin in saline) into the plantar surface of the right hindpaw. The total time spent in licking and flinching of the injected paw was monitored for 60 min at 5-min intervals.

### Assessment of motor function

A rotarod system of accelerating treadmills (Ugo Basile, Italy) was used to assess coordinate motor activity and general motor disability as described [27]. The mice were tested for 3 trials with 15-min intervals.

### Formalin-induced conditioned place avoidance (F-CPA)

This procedure was modified from F-CPA in rats [6,7]. Animals were trained in a shuttle box (47.5 × 20 × 20 cm^3^), containing three compartments separated by guillotine doors. Two large conditioning compartments (A and B, 20 × 20 × 20 cm^3^) were separated by a small gray center choice compartment (C, 7.5 × 20 × 20 cm^3^). The A compartment had white walls and stiff metal mesh flooring with an odor of 1.0% acetic acid, and the B compartment had black walls and parallel metal bars flooring with cinnamon scent. The C compartment had gray walls and a plain floor without distinctive odor. The experimental procedures were recorded by a video camera connected to a computer. The training procedure lasted for 5 days. The pre-conditioning test was performed on Day 1. Mice were placed into the central compartment and allowed to freely explore the entire apparatus for 20 min. Time spent in each compartment and the travel distance in three compartments was recorded. Any animal that spent more than 600 sec or less than 300 sec in any large compartment will be discarded from the experiment. Only animals that did not show a baseline preference were admitted into the study (>90% mice met this criteria). Then, mice were conditioned for 3 consecutive days with 2 pairing sessions each day. In the first session, the animals were restricted to one of the conditioning compartments (50% of the mice in compartment A, 50% of them in compartment B) for 35 min in the morning. In the second session in the afternoon, about 4 hr later, the animals received hindpaw injection of 5% formalin (15 μl) or normal saline (15 μl), and restricted to the opposite conditioning compartment for 35 min. For post-conditioning test on day 5, the animals were tested as day 1. On both day 1 and day 5, the animals' activities were recorded by a wide-angle video camera (Creative Technology Ltd.) connected with a computer, and the travel distance and time in each compartment were analyzed by ANYMAZE software (Stoelting Co., Wood Dale, Illinois USA).

### LiCl-induced conditioned place aversion (LiCl-CPA)

The apparatus for LiCl-CPA training was identical to that in F-CPA. The training procedure was also similar to that of F-CPA. In brief, the training procedure lasted for 5 days. On day 1, mice were placed into the central compartment and allowed to freely explore the entire apparatus for 20 min. Time spent in each compartment and the travel distance in three compartments was recorded. Animals showing baseline preference were discarded from the experiment. On day 2–4, mice were conditioned for 3 consecutive days with 2 pairing sessions each day. In the first session, the animals received saline (10 ml/kg, i.p.) and restricted to one of the conditioning compartments for 35 min in the morning. In the second session in the afternoon, the animals received LiCl (150 mg/kg, i.p.) and restricted to the opposite conditioning compartment for 35 min. On day 5, the animals were tested as day 1. On both day 1 and day 5, the animals' activities were recorded. The travel distance and time in each compartment were analyzed.

### *In situ *hybridization

Mice were anesthetized with sodium pentobarbital (50 mg/kg i.p.) and euthanized by transcardiac perfusion (saline wash, followed by 4% paraformaldehyde in 0.01 M phosphate buffer saline pH 7.4). The mouse brain was removed and post-fixed for 4 hr, then stored in 0.01 M PBS containing 30% sucrose for at least 24 hr for cryoprotection. Thin sections were made using an RNase-free technique. *In situ *hybridization was performed as described [25].

### Statistical analysis

Statistical comparisons were performed with two-way analysis of variance (ANOVA) or Student's *t*-test. Data are shown as mean ± S.E.M. (standard Error of Mean) and error bars represent S.E.M. In all cases, *P *< 0.05 was considered statistically significant.

## Results

### Identification of the ACC-enriched genes

To study the molecular mechanism of pain affection, we have searched the Allen Brain Atlas expression database [24] for genes whose expression is higher in the ACC (Table 1). Three genes, Marcksl1, P311, Etv1, showed the restricted expression pattern in the ACC.

**Figure 1:**
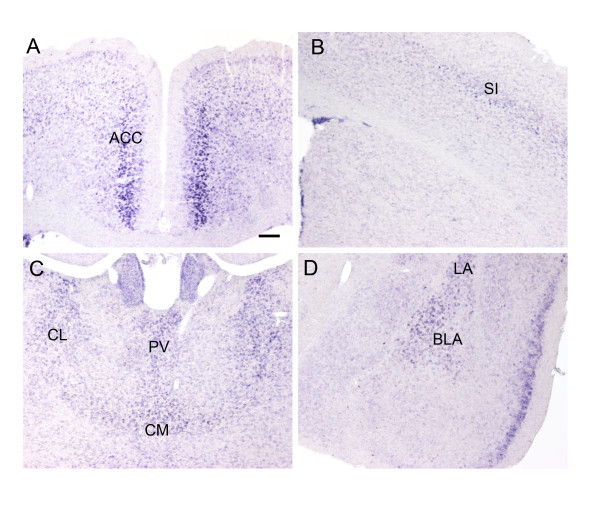
**Expression pattern of P311 in mouse brain detected by *in situ *hybridization**. **A**. P311 is expressed in the anterior cingulate cortex (ACC). **B**. Weak expression of P311 was detected in the somatosensory cortex (SI). **C**. P311 is also expressed in the centrolateral thalamic nucleus (CL), the central medial thalamic nucleus (CM) and the paraventricular thalamic nucleus (PV). **D**. In the amygdala, P311 is expressed in the basolateral amygdala nucleus (BLA) and the lateral amygdala nucleus (LA). Scale bar: **A**. 100 μm (**A-D**).

*In situ *hybridization studies indicated that P311 was abundantly expressed in the ACC as well as in other pain affection related regions of the brain (Fig. 1A, 1C, 1D, data not shown) [2,7]. P311 was weakly detected in the somatosensory cortex and dorsal spinal cord (Fig. 1B, data not shown). Expression of P311 was also detected in the hippocampus and cerebellum, which is consistent with the previous report [18]. P311 expression was strong in the rostral ventromedial medulla (data not shown), but not detected in the hypothalamus. Despite its extensive expression in the brain, the function of P311 in the nervous system remains largely unknown.

### Effects of the P311 mutation on the sensory component of pain perception

**Figure 2:**
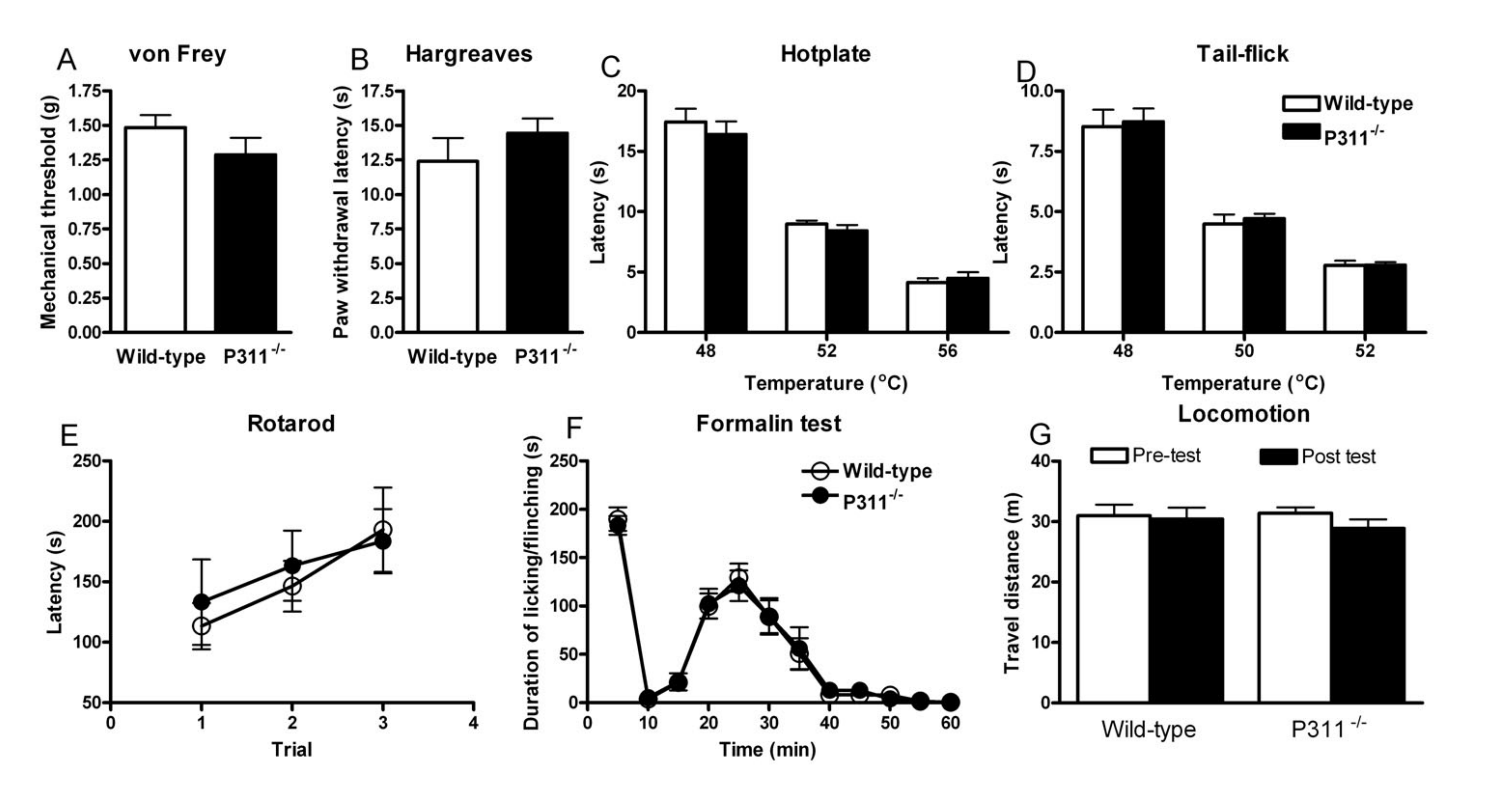
**Normal pain behaviors and locomotor activity in P311^-/- ^mice**. **A**. Acute pain measured by mechanical threshold was comparable between wild-type (*n *= 8; white bars) and P311^-/- ^mice (*n *= 9; black bars). **B-D**. Responses to noxious thermal stimuli were measured by the paw withdrawal latency to Hargreaves test (**B**), hotplate (**C**) and the water immersion tail-flick latency (**D**). Thermal pain in all tests did not differ between wild-type (*n *= 8; white bars) and P311^-/- ^mice (*n *= 9; black bars). Student's *t*-test, *P *> 0.05. **E**. Motor function assessed by the rotarod test was not affected in P311^-/- ^mice (*n *= 9; filled circles) compared with wild-type mice (*n *= 8; open circles). Repeated measures analysis of variance; *P *> 0.05. **F**. Spontaneous pain responses induced by formalin were comparable between wild-type (*n *= 9; open circles) and P311^-/- ^mice (*n *= 10; filled circles). Repeated measures analysis of variance; *P *> 0.05, Phase I (0–10 min); *P *> 0.05, Phase II (10–60 min). **G**. Travel distance was monitored during the pre-test (day 1) and post-test day (day 5) of experiment by the ANYMAZE software. There was no significant difference between wild-type and P311^-/- ^mice on both the pre-test and post-test day. And the travel distance was not affected by formalin injection in either wild-type or P311^-/- ^mice. *n *= 16 for each group. Student's *t*-test, *P *> 0.05.

Because P311 is expressed in the sensory pathway of pain, we first assessed the role of P311 in pain by comparing thermal, mechanical and inflammatory pain responses of P311^-/- ^mice with their wild-type littermates [25]. We found that the latencies responding to noxious thermal stimuli in Hargreaves test, hotplate, and tail flick test as well as mechanical stimuli produced by graded von Frey filaments were indistinguishable between P311^-/- ^and wild-type mice (Fig. 2A–D). P311^-/- ^mice exhibited normal motor function (Fig. 2E). In formalin test, flinching and licking behaviors in both the first phase (0–10 min) and the second phase (10–60 min) were also comparable between P311^-/- ^and wild-type mice (Fig. 2F), indicating that a loss of P311 does not impact normal inflammatory pain response. Together, these data suggest that P311 is not essential for the sensory component of pain perception.

### Effects of the P311 mutation on the affective component of pain

**Figure 3:**
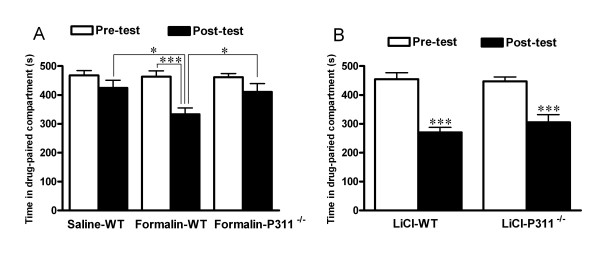
**Defect of pain affection in P311^-/- ^mice**. Time spent in each compartment was monitored during the pre-test (day 1) and post-test day (day 5) of the experiments.** A**. In wild-type mice, formalin (*n *= 16) but not saline (*n *= 12) induced conditioned place avoidance. Conditioned place avoidance was induced by formalin in wild-type mice (*n *= 16), but not in P311^-/- ^mice (*n *= 16). Student's *t*-test, *P < 0.05, ***P < 0.001. **B**. LiCl (150 mg/kg, i.p.) induced conditioned place aversion in both wild-type (*n *= 10) and P311^-/- ^mice (*n *= 10) in a comparable manner. Student's *t*-test, ***P < 0.001.

We next tested the requirement of P311 in the affective component of pain by F-CPA, which is a well-established model for testing pain affection in rats [6,7]. When hindpaw formalin injections were paired with a particular compartment in the place-conditioning apparatus, wild-type mice spent significantly less time in this compartment on the post-conditioning test day as compared with the preconditioning test day (Fig. 3A). In the saline-treated group, animals showed no significant avoidance to the conditioned environment (Fig. 3A). The time spent in the treatment-paired compartment of formalin-treated mice was significantly less than that of saline-treated mice on post-test day (Fig. 3A). These results validated the utility of the F-CPA model in mice. When the same training procedure was used in P311^-/- ^mice, CPA was not induced (Fig. 3A). The finding that P311^-/- ^mice, unlike their littermates, displayed no aversive behaviors to formalin injections suggests that P311 is important for the acquisition or expression of F-CPA. In addition, the travel distance on both pre-test and post-test day was comparable between wild-type and P311^-/- ^mice (Fig. 2G), suggesting that lack of F-CPA in P311^-/- ^mice was not caused by a change in locomotor activity.

### LiCl-CPA was not affected by the absence of P311

To distinguish whether lack of aversive behavior in P311^-/- ^mice was confounded by a deficit in associative learning and memory, we next examined aversive learning task of P311^-/- ^and wild-type mice by using the LiCl-CPA paradigm [28]. LiCl induced CPA in both wild-type and P311^-/- ^mice (Fig. 3B), suggesting that the P311 mutation did not reduce the animal's ability to acquire a CPA when the stimulus was unpainfully aversive. This result further suggests that the absence of P311 did not impact the animal's ability to associate the aversive stimulus with the distinct environmental context. Therefore, we conclude that the absence of P311 does not alter the learning ability in the place-conditioning paradigm, but rather results in a deficit concerning the acquisition or expression of pain-related aversion.

## Discussion

In this study, we have identified several genes including P311 which are highly enriched in the ACC. We set out to examine whether P311 is important for pain perception. Despite P311 is also expressed in neural pathways required for mediating sensory component of pain, we found that mice lacking p311 showed normal acute and persistent pain behaviors in several pain paradigms. Remarkably, P311^-/- ^mice showed deficits in pain affection assessed by the F-CPA paradigm.

Our finding that the P311 mutation abolished CPA induced by formalin suggests that P311 is important for the aversion behavior induced by painful stimuli. Since it is well accepted that the avoidance behavior associated with noxious stimuli can serve as a reflex of affective component of pain, it is reasonable to believe that P311 is necessary for the acquisition or expression of avoidance behavior induced by the noxious stimulus, and thus pain affection. Importantly, this compromised affective pain in the mutant mice is not due to a deficit in associate learning and memory. This is evidenced by the result of the LiCl-CPA paradigm, in which CPA was induced at a similar manner in both wild-type and P311^-/- ^mice. This result further indicates that P311 is not involved in the aversion behavior associated with non-painful stimuli. Although P311 is also expressed in the rostral ventromedial medulla and the dorsal spinal cord, the findings that the pain behaviors of P311^-/- ^mice were normal in several thermal, mechanical and chemical pain paradigms suggest that P311 is dispensable for the sensory processing of pain.

Involvement of P311 in pain affection might not be restricted to the ACC since P311 is also expressed in the regions important for pain affection such as the amygdala, and the thalamus (mainly in the medial/intralaminar thalamic nuclei) [2]. In addition to its role in fear conditioning [29,30], the amygdala has also been implicated in processing pain affect. Bilateral lesion of the amygdala significantly reduced conditioned response in the laser-pain conditioning model [31] and F-CPA model [7,32] without affecting normal behavior or baseline nociceptive responses, suggestive of an involvement of the amygdala in pain affection. The medial/intralaminar thalamic nuclei which relay the information from the dorsal spinal cord to the ACC and the insular cortex has been proposed to process pain-related unpleasantness [2,33,34]. Although there is no direct evidence from animal experiments showing the involvement of the insular cortex in pain affect, the widespread connections among the insular cortex, thalamus, amygdala, hippocampus and cortical regions related to sensory modalities and autonomic functions suggest the involvement of the insular cortex in autonomic reactions to noxious stimuli and in pain-related learning and memory [2,35]. Given the presence of P311 in these areas, it is unclear if P311 is required for processing the affective dimension of pain in all of the regions or only in one particular area. Future experiments such as the site-specific deletion of P311 or overexpression of P311 are necessary to determine the site of P311 deficiency that may account for the deficit in pain affection.

The mechanism by which P311 exerts its function in pain affection might involve the modulation of strength of the neural circuits processing or storing affective pain information. There are at least two possibilities. First, P311 may modulate the neuroplasticity by remodeling the spine of the neurons. For example, activity-dependent structural remodeling of dendritic spines in the cortex has been shown to be important for LTP and Long-term depression [36,37], and has been postulated as a cellular basis of learning and memory [38]. In this regard, recent work indicating that P311 can promote neurite outgrowth of postnatal neurons [20], possibly by the reorganization of cytoskeleton [39] is of interest. Second, P311 may modulate the neural activity in the ACC. Recent studies have shown that NMDA and AMPA receptors are important for pain processing in the ACC [10,15], so it is likely that P311 may regulate the neuronal activity by directly or indirectly interacting with the excitatory receptors. Nevertheless, the actual mechanism by which P311 regulates the affective pain remains to be explored.

## Conclusion

Regardless of mechanisms involved, our findings suggest for the first time that sensory and affective pain may be dissociated from each other at the molecular level. To our best knowledge, our report represents the first to suggest that a unique set of genes may be required for the function of neural circuits underlying the pain affection, thereby providing the molecular logic for explaining the partially dissociable brain networks which are responsible for two distinct components of pain perception [2,3]. Identification of genes involved in the affective but not in the sensory component of pain may have therapeutic value in the management of pain affection. Since P311 is highly conserved between the rodents and human and formalin injection represents a noxious stimuli to human [18,40], it is conceivable that P311 may be a potential target for alleviating the affective component of pain.

## Competing interests

The authors declare that they have no competing interests.

## Authors' contributions

YGS performed pain behavioral experiments and *in situ *hybridization studies, YJG performed F-CPA and LiCl-CPA experiments, ZQZ and BH contributed to CPA experiment, YJ performed genotyping of mice, GAT provided P311 mutant mice, ZFC, YJG and YGS prepared the manuscript. All authors read and approved the manuscript.
